# Selective Detection of Liquid Viscosity Using Acoustic Plate Waves with In-Plane Polarization

**DOI:** 10.3390/s22072727

**Published:** 2022-04-01

**Authors:** Vladimir Anisimkin, Elizaveta Shamsutdinova, Peng Li, Bin Wang, Feng Zhu, Zhenghua Qian, Iren Kuznetsova

**Affiliations:** 1 Kotelnikov Institute of Radio Engineering and Electronics of RAS, 125009 Moscow, Russia; anis@cplire.ru (V.A.); shes1996@bk.ru (E.S.); 2 State Key Laboratory of Mechanics and Control of Mechanical Structures, College of Aerospace Engineering, Nanjing University of Aeronautics and Astronautics, Nanjing 210016, China; lipeng_mech@nuaa.edu.cn (P.L.); wangbin1982@nuaa.edu.cn (B.W.); zhufeng.phd@nuaa.edu.cn (F.Z.)

**Keywords:** liquid viscosity, conductivity, temperature, piezoelectric plate, acoustic plate wave, attenuation

## Abstract

Using plates of weak piezoeletcric crystal (quartz) loaded with various liquids, it is shown that along with common modes, whose sensitivity towards different liquid parameters comparable with each other, there are some uncommon modes, whose amplitude responses towards viscosity η are much larger than towards temperature T and electric conductivity σ. The search of the modes with the selective properties is accomplished by varying plate thickness h, crystal orientation, wave length λ, and mode order n. It is found that all modes possessing the property are characterized by small surface-normal displacement, avoiding wave radiation into adjacent liquid, large in-plane displacements, enhancing viscous coupling the modes and liquids, and small electro-mechanical constant, reducing electro-acoustic interaction. Basing on the modes, the sensor prototypes with selective operation are developed and tested for η from 1 to 1500 cP, σ from 0 to 1.2 S/m, and t from 0 to 55 °C. Because of operation at ultrasonic frequency (tens MHz) the prototypes have different sensitivities in various η-ranges: 0.3 dB/cP for 1–20 cP, 0.12 dB/cP for 20–100 cP, and 0.015 dB/cP for 100–1500 cP. Viscosity responses of the prototypes become comparable with their electric outputs only for η < 2 cP. Temperature responses are almost zero in air, but when plate is coated with liquid they increase depending on liquid properties, allowing measurements of the temperature dependence of the liquid viscosity.

## 1. Introduction

The measurement of fluid viscosity is an important problem for various applications in some industries (machine, instrument, chemical, food, pharmaceutical), agriculture, bio-engineering, ecology, etc. [1,2,3,4,5,6,7]. The knowledge on viscosity is also necessary in medicine for characterizing rheological bio-liquids (blood, sperm) [8]. To measure this parameter a need was recognized for microsensing devices with small size, high precision, good reliability, and multiple usages. One of the most attractive approaches for developing such sensors is the use of acoustic wave propagation [9]. Acoustic devices are relay upon the changes in the wave amplitude and velocity when a sample of liquid loads propagation medium [10]. The acoustic sensors are potentially attractive because they do not require large fluid samples, do not introduce significant distortions into the probe, allow remote data collection via radio channel, and have two independent responses (phase and amplitude) to each action. Performance of the sensors depends evidently on the type of acoustic wave exploited in device. Till know Rayleigh surface acoustic waves (SAW) [10,11], shear-horizontal (SH) SAW [12,13,14,15], leaky SAW [16], bulk acoustic waves [17,18,19,20], slot acoustic wave [21], SH plate acoustic waves (PAW) of zero order [22], and PAW of higher order propagating in isotropic [23,24] and piezoelectric [25,26,27,28,29,30] and plates were used for the purpose. The resonators with a lateral electric field excitation have recently been suggested for measurement conductivity and viscosity of liquids [31]. For this purpose it is possible to use new structures like phononic crystals with various periodic inclusions as well [32,33,34].

Recently, new modification of the Lamb waves was found [35]. It has small vertical displacement accompanied with large shear-horizontal and longitudinal components, i.e., elliptic polarization parallel to the plate faces. The modified wave generated at 49.74 MHz in ST,X-quartz plate with normalized plate thickness h/λ = 1.0 (h—thickness, λ—wave length) responds strongly on glycerin loading (viscosity η = 1490 cP), thereby demonstrating high sensitivity towards fluid viscosity.

In general, most acoustic sensors are based on common delay lines with input and output interdigital transducers (IDTs) and test liquid located between them. Usually, most modes propagating in crystal plates with liquid loadings are sensitive to different liquid parameters more or less equally [9]. As a result, in order to measure a single parameter alone, special sensor configurations, several independent measurements, and relevant signal processing are required [29,30]. This property brings up the question: are there acoustic waves, whose partial response towards one liquid parameter (e.g., viscosity η) is much larger than towards others (e.g., temperature T and electric conductivity σ). If so, selectivity of the wave will provide direct measuring relevant liquid parameter without any special configurations and signal processing.

The goal of the present paper is to study this question basing on quartz plates and various acoustic plate modes including new modification. Theoretical and experimental analysis of the waves is accomplished for fluids with different viscosities and electric conductivities.

## 2. Materials and Methods

### 2.1. Theoretical Methods

**Figure 1:**
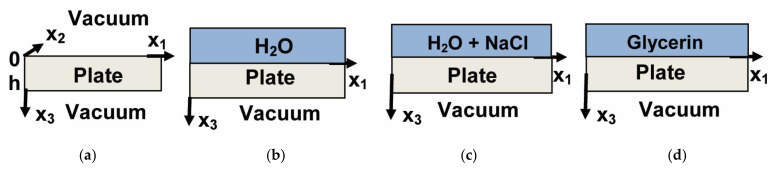
Geometry of the problems: structure “vacuum-plate-vacuum” (**a**), structure “vacuum-plate-H_2_O” (**b**), structure “vacuum-plate- water solution with NaCl” (**c**), and structure “vacuum-plate-glycerin” (**d**).

Let us consider an acoustic wave, propagating along x_1_ direction in a piezoelectric plate (Figure 1a). In order to find phase velocity and mechanical displacements of the wave the equation of motion, Laplace’s equation, material equations for electric displacement and mechanical stress are used for plate medium, while Laplace’s equation and equation for electric induction are used for vacuum. Then, relevant electric and mechanical conditions on vacuum/plate boundary are considered, and boundary condition matrix was formed. The value of wave velocity v_n_ is found using iterative search procedure by zeroing determinant of the matrix. After that three partial components of mechanical displacement (u_1_, u_2_, u_3_) are calculated for as-found value of velocity v_n_ at any depth x_3_ of the plate [36].

When a plate is loaded with semi-infinite not viscose, not conductive liquid (Figure 1b) the equation of motion, Laplace’s equation, material equations for electric displacement and mechanical stress are used for the liquid. The liquid/plate boundary conditions are the continuity of the normal mechanical displacement (u_3_^pl^ = u_3_^lq^), normal mechanical stress (T_33_^pl^ = T_33_^lq^), electric displacement (D_33_^pl^ = D_33_^lq^), and electric potential (Φ^pl^ = Φ^lq^) [37].

When a plate is loaded with semi-infinite non viscose, but conductive liquid (Figure 1c) Poisson’s equation instead of Laplace’s equation is used for liquid, while equation for continuity of electric charge, material equation for current, and condition of zero electric current are added to the plate/liquid boundary conditions [22].

When a plate is loaded with semi-infinite viscose, but nonconductive liquid (Figure 1d) Laplace’s equation instead of Poisson’s equation is used, while continuity of all mechanical displacements, mechanical stress, electric displacement, and electric potential are taken as boundary conditions. In this case, viscosity of the liquid is accounted as imaginary part *i*ωη_ij_ of elastic moduli, where *i* is imaginary value, ω = 2πf is circular frequency, η_ij_ are viscosity coefficients in Pa × s [22].

Material constants of quartz, distilled H_2_O, and glycerin are taken from [38,39,40] and presented in Table 1.

### 2.2. Experimental Methods

**Figure 2:**
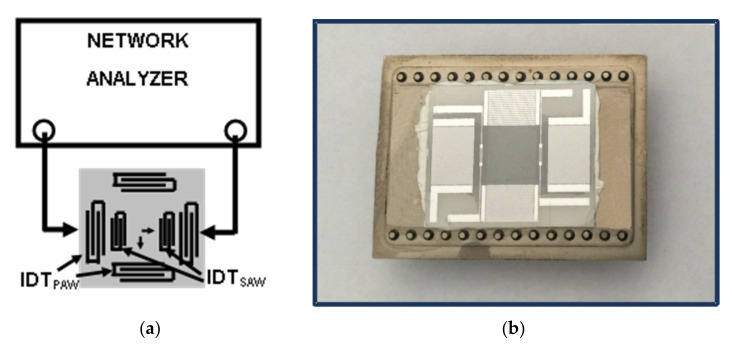
Schematic view (**a**) and photo (**b**) of a test sample with interdigital transducers generating Lamb and SH (PAW), and surface (SAW) acoustic waves.

The measurements are carried out at room temperature and atmospheric pressure. The plates of ST-quartz with Euler angles 0°, 132.75°, 0° and 0°, 132.75°, 90° are used as a weak piezoelectric material. The normalized thickness of the plates are h/λ = 0.6, 1.0, 1.25, 1.485, and 1.67 (h = 300 and 500 μm, λ = 200, 202, 300, 400 and 500 μm), providing variety of the modes for analysis [35,41,42]. The plates have one grinded (top) and one polished (bottom) surface. The top surface is sealed with a liquid cell (fused quartz) which is large enough to avoid perturbation of an acoustic beam by the cell. The bottom surface contains three pairs of interdigital transducers (IDTs) having periodical structure. Two pairs of identical transducers with periods λ = 200, 202, 300, 400 or 500 μm are aligned perpendicular each other (Figure 2a). Details of the test samples which are sufficient for reproducing experimental results are presented in Table 2. The 1st pair is used to generate generalized Lamb modes along X-axis (Euler angles 0°, 132.75°, 0°); the 2nd pair excites shear-horizontal (SH) modes perpendicular to X-axis (0°, 132.75°, 90°); the 3rd pair of small transducers with period λ = 20 μm is intended for controlling the plate and liquid temperature T by surface acoustic waves: the change ΔT, if any, is detected as ΔT = (TCD)^−1^ × Δφ/φ, where Δφ/φ is the change in the phase of the wave and TCD is the wave temperature coefficient [43]. Each transducer comprises of 20 finger electrodes patterned from 1000-nm-thick Cr/Al. The large number of electrodes provides good frequency resolution for neighboring acoustic plate modes with close velocities v_n_. 

**Figure 3:**
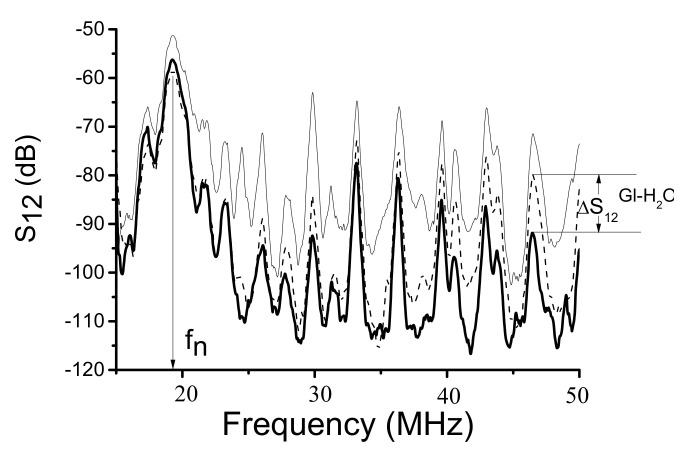
Typical insertion loss S_12_ measured in ST,X-quartz plate with two free faces (thin solid), one free face and one water-loaded face (dashed), and one free face and one glycerin-loaded face (thick solid). Plate: h = 500 μm, IDTs: λ = 300 μm, h/λ = 1.67. Liquids are over the propagation path L = 22 mm on the bottom of the plate.

The measurements of the insertion loss S_12_(f) are carried out using KEYSIGHT 5061B network analyzer (Keysight, Santa Rosa, CA, USA) operating in amplitude-frequency format (Figure 2a). In order to avoid an influence of electromagnetic leakage the amplitude-frequency format S_12_(f) is converted to the amplitude-time format S_12_(τ), where the gate window is started just after the leakage and stopped after acoustic signal. When the gate is on, the leakage is off and the time delay format S_12_(τ) converted back to the frequency format S_12_(f) without the leakage (Figure 3).

The mode velocities are measured as v_n_ = λ × f_n_, where λ is period of IDTs (the wavelength), f_n_ is the central frequency of the modes (Figure 3). Precision of the measurements is ±1%.

The measurement of the attenuation coefficient α_n_ produced by a liquid is carried out also using KEYSIGHT 5061B network analyzer (Keysight, Santa Rosa, CA, USA) (Figure 2). For each mode n the value of the insertion loss S_12_^air^ is, first, measured in air (without liquid) at relevant frequency f_n_ = v_n_/λ (Figure 3). Second, the same loss S_12_^H^_2_^O^, S_12_^H^_2_^O+Gl^, and S_12_^Gl^ are recorded after distilled water (viscosity η = 1.003 cP), water solutions of glycerin (1.003 cP <η < 1491 cP) and pure glycerin (η = 1491 cP) are introduced into the cell one by one as liquids with variable viscosity, zero electric conductivity σ = 0 and slightly varied density (<26%) and permittivity (<10.5%) [39]. Third, the attenuation coefficient α_n_ for each mode and test liquid is deduced as α_n_ = (S_12_^Gl^ − S_12_^H^_2_^O^)/L = ΔS_12_^(Gl-H^_2_^O)^/L, where L is a propagation path of a mode along a liquid. Finally, for each sample the modes n with largest α_n_ are determined and compared with one another. Precisions of the measurements are ±0.01 dB for S_12_ and ±0.005 dB/mm for α_n_. 

In order to measure sensitivity of the modes towards liquid conductivity σ the water solutions of NaCl with σ varied from 0 (distilled water) to 10 S/m (7.6 weight % NaCl in water) are used as test liquids with almost constant viscosity (<13%), density (<8%), and permittivity (<1%) [39]. 

Taking into account strong dependence of liquid properties on the temperature T, most measurements are carried out at 20 ± 0.1 °C fixed by thermal camera UC-20CE (NOSELAB ATS, Nova Milanese, Italy). On the other hand, in order to study the temperature sensitivity of the sensor the camera is heated from T = 0 to 55 °C with the step ΔT = 5 °C and same measurements for each temperature are accoplished without any liquid and with water and glycerin. When a liquid is absent the sensor slightly responds to temperature according to properties of the wave (S_12_^air^~0.1 dB). When a liquid is present, the response of the sensor is additionally depends on the value of liquid viscosity at relevant temperature. So that, by extracting 1st set of data from the 2nd gives us the temperature dependence of viscosity η(T) for a given liquid. Precision of the measurements is ±20%.

All liquid solutions are prepared by mixing partial components in forced vibrator for about 5 min. Knowing the components weights, the values η and σ at 20 °C are found from [39]. An error in weight concentration of glycerin and NaCl in water is about ±1%.

The volumes of the test liquids sufficient for making measurements are about 100 µL.

## 3. Results and Discussion

Table 3 shows results of the calculations for higher-order modes propagating in quartz plates with two free faces and plates with one free, one liquid loaded face. It is seen that (i) mode 1 of the Table 3 has dominant u_3_^0^ component (u_3_^0^>> u_1_^0^, u_2_^0^) and, thereby, large radiation loss into the liquid; (ii) modes 3 and 4 possess dominant shear-horizontal components (u_2_^0^ >> u_1_^0^, u_3_^0^) and, therefore, the attenuation of the modes is not originated from radiation, but from relaxation process related with liquid viscosity. The modes of this type has recently been used for viscosity sensors [22,25,26,27]; (iii) mode 2 has small vertical displacement, large shear-horizontal and large longitudinal components, i.e., elliptic polarization which is oriented parallel to the plate faces (u_1_^0^, u_2_^0^ >> u_3_^0^), the polarization being maintained for any loading. So that, the mode 2 is just the modified Lamb wave [35]. The attenuation of the mode is originated from viscous coupling. The electromechanical coupling coefficient of the mode 2 is small (k^2^ = 0.02%).

Total amount of modes detected in 9 quartz plates is as large as 100. Most of them are not suited for application because of too large attenuation arising from (i) compression wave radiation into adjacent liquid (proportional to surface-normal component u_3_), and (ii) viscous coupling the modes and liquid (proportional to in-plane components u_1_ and u_2_). Nevertheless, the modes with allowable attenuation are found in the plates though the liquid-loaded loss for some modes approaches 100 dB (Table 4, last column).

Best modes belonging to SH and Lamb families and propagating in different plates are presented in Table 4. The mode responses towards glycerin referred to responses for water are as large as Δα_n_^(Gl-H_2_O)^ = 1.08–1.64 dB/mm (bold).

**Figure 4:**
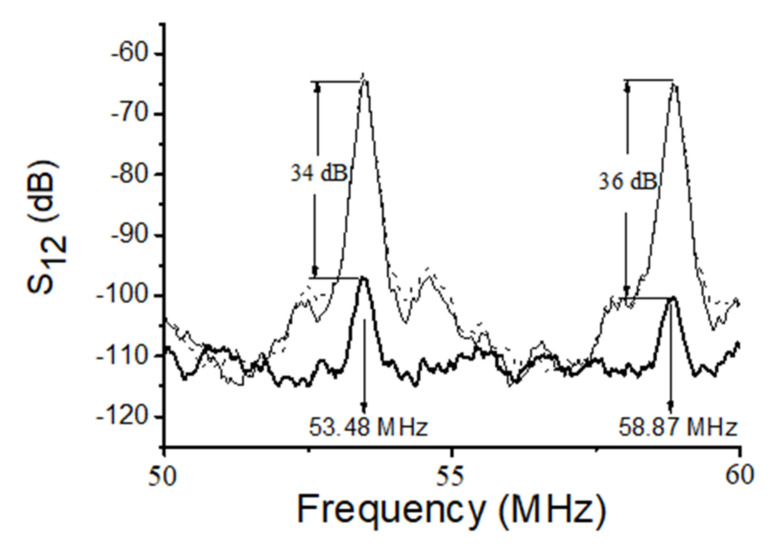
Insertion loss S_12_ of the SH-modes with high sensitivity towards viscosity measured at h = 300 μm, λ = 300 μm, and h/λ = 1.0 in ST,X + 90°-quartz plate with two free faces (thin solid), one free face, one water-loaded face (dashed), and one glycerin-loaded face (thick solid). Liquids are over the propagation path L = 22 mm on the bottom of the plate.

**Figure 5:**
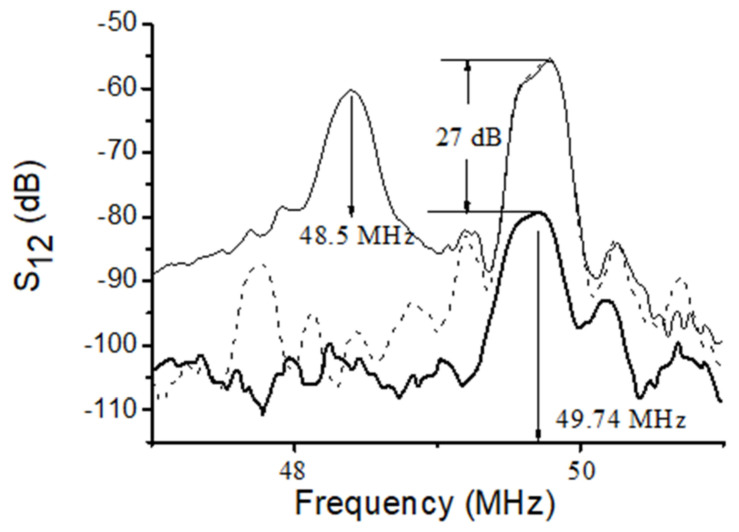
Insertion loss S_12_ of the Lamb modes with large radiation loss (48.5 MHz) and with high sensitivity towards viscosity (49.74 MHz) measured at h = 300 μm, λ = 300 μm, and h/λ = 1.0 in ST,X-quartz plate with two free faces (thin solid), one water-loaded face (dashed), and one glycerin-loaded face (thick solid). Liquids are over the propagation path L = 22 mm on the bottom of the plate.

Examples of the best modes are shown on Figure 4 (f_n_ = 53.48 and 58.87 MHz) and Figure 5 (f_n_ = 49.74 MHz). The properties of the modes measured experimentally (Figure 4 and Figure 5) are in agreement with those calculated numerically (Table 3). Indeed, having negligible normal component u_3_, eliminating compression wave radiation into adjacent liquid, the loss S_12_ measured for these modes with water loading (dashed) and without any liquid (solid) are almost equal each other (S_12_^H^_2_^O^ ≈ S_12_^air^). Also, having large in-plane components (u_2_ or u_1_ and u_2_), the same modes have high sensitivity towards viscosity (bold). Other modes (e.g., the mode detected at 48.5 MHz, Figure 5) have large normal component (u_3_/u_1_ = 130) and big radiation loss (>40 dB). These modes are useless for application.

**Figure 6:**
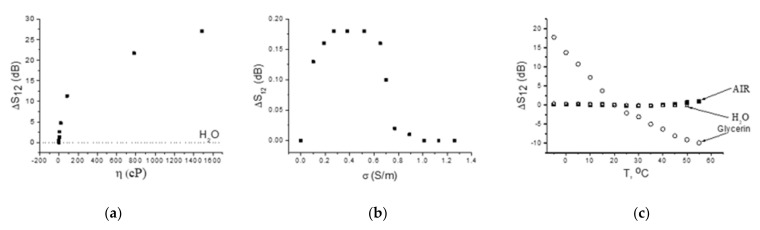
Calibration curves of the sensor prototype for viscosity (**a**), electric conductivity (**b**), and temperature (**c**) for ST,X-quartz plate, h/λ = 1.0, mode frequency 49.74 MHz. Liquids are over the propagation path L = 22 mm on the bottom of the plate.

Some modes from Table 4 are utilized for developing sensor prototypes. The calibration corves for one of them are shown on Figure 6. The curve for viscosity (Figure 6a) is typical for waves vibrating at ultrasonic frequencies [10]: it is almost linear for small η, when liquid behaves as ideal (Newtonian), and saturates for large η, when liquid behaves as a solid [10]. As a result, the sensitivity of the sensor is varied from 0.3 dB/cP for η = 1–20 cP to 0.12 dB/cP for η = 20–100 cP and 0.015 dB/cP for η = 100–1500 cP.

The calibration curve of the same sensor for conductivity σ (Figure 6b) is also typical [22]: the electric response increases for small σ < 0.4 S/m, approaches maximum at σ = 0.4 S/m, and falls down to zero for large σ > 1 S/m, when sensor becomes insensitive to liquid conductivity. Moreover, as the coupling coefficients of the modes from the Table 3 are small (<0.02%), the largest electric response (Figure 6b) is much lower than that is for viscosity (Figure 6a). The two responses become comparable with each other only for liquids with very small viscosities η < 2 cP.

**Figure 7:**
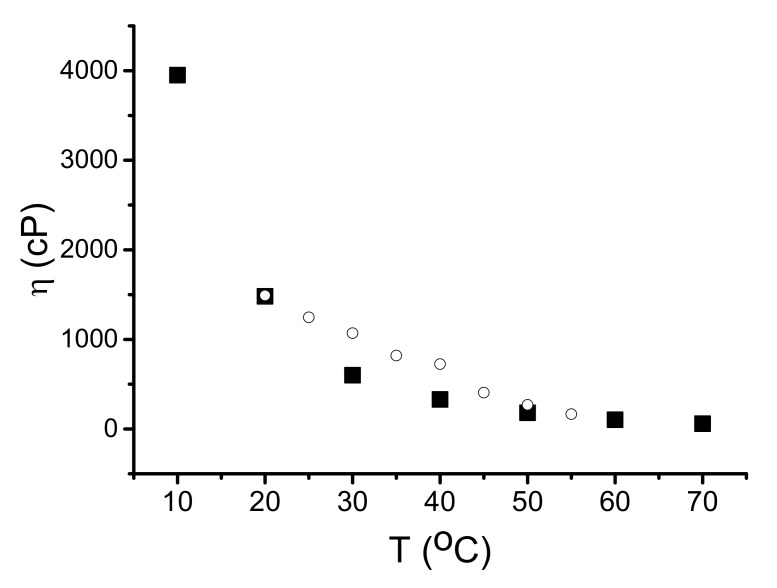
Temperature dependence of glycerin viscosity measured in the paper (circulars) by using Lamb mode with frequency 49.74 MHz in ST,X-quartz plate with h/λ = 1.0 and the same dependence taken from [39] (squares). Precision of our measurements is ±20%.

The temperature sensitivity of the same prototype without liquid loading (AIR) is negligible, but when a liquid is present, the prototype responds to the temperature accordingly to temperature dependence of the liquid viscosity (Figure 6c). Extracting the data for unloaded plate from the data for plate with test liquid allows measuring the temperature dependence of viscosity η(T) for the liquid. As an example, Figure 7 shows the as-measured (open rings) and the tabulated (black squares [39]) data for glycerin. It is seen both data are in agreement with one another.

The view of sensing element located in holder is shown on Figure 2b. Three pairs of interdigital transducers located on the bottom of quartz plate are visible through the plate thickness. Test liquid is deposited in central part of the top surface between transducers.

## 4. Conclusions

Modified Lamb and SH acoustic plate waves with increased sensitivity to liquid viscosity together with decreased sensitivity to liquid conductivity and temperature are found in quartz plates with thickness h of about wavelength λ. The modes are characterized by small or zero surface-normal displacement, avoiding mode radiation into adjacent liquid, and by large in-plane displacements, enhancing viscous coupling the wave and liquid deposited on the plate.

Basing on the waves with in-plane polarization, selective viscosity sensors are developed: sensitivity of the sensors towards viscosity is 0.3 dB/cP for 1–20 cP, 0.12 dB/cP for 20–100 cP, and 0.015 dB/cP for 100–1500 cP; responses towards conductivity (0 to 2 S/m) are two orders of magnitude smaller; temperature responses are almost zero in air, but when plate is coated with liquid they increase depending on liquid properties. Temperature dependence of glycerin viscosity measured by the sensor is in agreement with published data [39].

## Figures and Tables

**Table 1:** Density ρ (kg/m^3^), elastic constants C_ij_ (GPa), piezoelectric coefficients e_ij_ (C/m^2^), viscosity coefficients η_ij_ (Pa × s) and dielectric permittivity ε_ij_/ε_0_ of quartz, distilled H_2_O and glycerin used in calculations (T = 22.5 °C).

Quartz													
**ρ**	**C^E^_11_**	**C^E^_12_**	**C^E^_13_**	**C^E^_14_**	**C^E^_24_**	**C^E^_33_**	**C^E^_44_**	**C^E^_66_**	**e_11_**	**e_12_**	**e_14_**	**ε_11_/ε_0_**	**ε_33_/ε_0_**
2650	86.7	7	11.9	−17.9	17.9	107	57.9	39.85	0.171	−0.171	−0.0406	4.4	4.6
**H_2_O**			**Glycerin**										
**ρ**	**C_11_**	**ε/ε_0_**	**ρ**	**C_11_**		**η_11_**		**C_44_**		**η_44_**		**ε/ε_0_**	
997.299	2.25	80	1260	2.81		118.6		1.2128 × 10^−3^		1.5		41.9	

**Table 2:** The characteristics of the test samples.

IDT Period λ, μm	IDT Aperture, μm	Number of Electrode Pairs	Face-to-Face Distance, μm
200, 202	5500	20	8500
300	9000	20	16,000
400	10,400	20	17,600
500	13,000	20	22,000
20	1600	20	8000

**Table 3:** The velocities v_ph_, normalized displacements u_1_^0^, u_2_^0^, u_3_^0^ at x_3_ = 0 and attenuation coefficients α_n_ of APM in quartz plates with two free faces and plates with one free–one liquid loaded faces. Plates: ST,X and ST,X + 90°-quartz with h/λ = 1. Loadings: distilled H_2_O, water solution of NaCl (conductivity σ = 0.24 S/m), and pure glycerin (viscosity η = 1491 cP).

#	Plate, Frequency	Free Plate v_n_, m/s (u_1_^0^; u_2_^0^; u_3_^0^) α_n_, dB/mm	Plate + H_2_O v_n_, m/s (u_1_^0^; u_2_^0^; u_3_^0^) α_n_, dB/mm	Plate + H_2_O + NaCl v_n_, m/s (u_1_^0^; u_2_^0^; u_3_^0^) α_n_, dB/mm	Plate + Glycerin v_n_, m/s (u_1_^0^; u_2_^0^; u_3_^0^) α_n_, dB/mm
1	ST,X-Quartz f_n_ = 48.5 MHz	14,529.277	14,528.546	14,528.52	disappeared
		(1; 9.2; 130)	(1; 2.8; 41)	(1; 2.9; 41)	
		0	1	1	
2	ST,X-Quartz f_n_ = 49.74 MHz	14,956.63	14,955.982	14,955.96	14,927.698
		(1; 1.6; 0.007)	(1; 1.6; 0)	(1; 1.6; 0)	(1; 1.6; 0.015)
		0	6.6 × 10^−6^	2.8 × 10^−4^	0.33
3	ST,X + 90°-Quartz f_n_ = 53.48 MHz	16,058.053	16,057.432	16,057.41	16,027.35
		(1; 77,000; 0.15)	(1; 77,381; 0.15)	(1; 77,427; 0.15)	(1; 75,689; 0.14)
		0	0	5 × 10^−5^	0.33
4	ST,X + 90°-Quartz f_n_ = 58.87 MHz	17,676.182	17,675.604	17,675.587	17,644.028
		(1; 68,700; 0.55)	(1; 72,613; 0.55)	(1; 72,893; 0.55)	(1; 52,615; 0.6)
		0	0	2 × 10^−4^	0.3

**Table 4:** Acoustic plate modes with largest responses towards viscosity measured in different quartz plates at 20 °C.

Plate	h/λ	f_n_, MHz	L, mm	ΔS_12_(Gl-H_2_O), dB	α_n_(Gl-H_2_O), dB/mm	S_12_(Gl), dB
ST,X-Quartz	0.6	19.95	20	**22.9**	**1.15**	**94.6**
ST,X + 90°-Quartz	0.6	19.88	20	10	0.5	74.2
ST,X-Quartz	1.0	49.74	22	**27**	**1.23**	**79.6**
ST,X + 90°-Quartz	1.0	53.48	22	**34**	**1.55**	**100.5**
		58.87	22	**36**	**1.64**	
ST,X + 90°-Quartz	1.25	34.26	30	13.9	0.46	89.4
ST,X-Quartz	1.485	28.85	31	23.6	0.76	72.1
ST,X + 90°-Quartz	1.485	46.36	31	**33.5**	**1.08**	**94**
ST,X-Quartz	1.67	47.1	22	17.5	0.8	−91.9
ST,X + 90°-Quartz	1.67	46.73	22	20	0.9	−81.3
